# Effects of Nanoparticles and Surface Modification on Thermal, Mechanical, and Electrical Properties of Composites from Liquid Silicone Rubber with Expanded Graphite

**DOI:** 10.1021/acsomega.4c10633

**Published:** 2025-04-15

**Authors:** Xingrong Liu, Zhaoyang Ma, Dietmar Auhl, Fan Xia

**Affiliations:** a Department of Polymer Materials and Technologies, Technische Universität (TU) Berlin, Ernst-Reuter-Platz 1, Berlin D-10587, Germany; b East China University of Science and Technology, Shanghai 200237, P.R. China

## Abstract

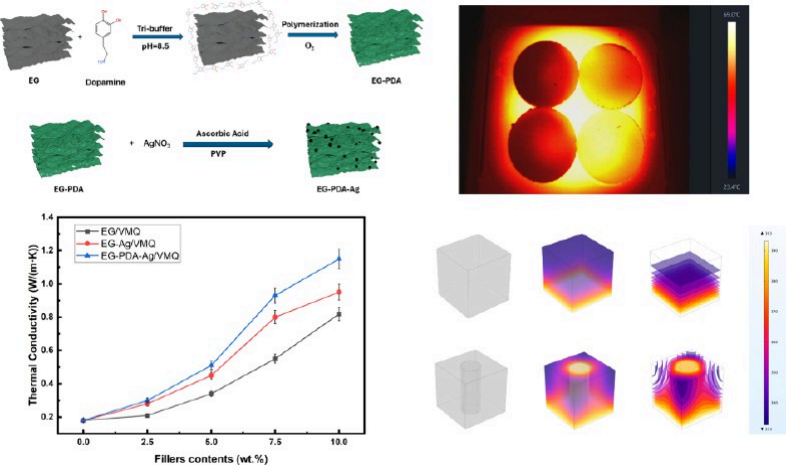

In this study, expanded graphite-poly(dopamine)-silver (EG-PDA-Ag) hybrid nanoparticles were synthesized in situ using a simple and environmentally friendly method. The EG nanoparticles were first PDA-modified by oxidative self-polymerization reaction of dopamine (PDA) in weakly alkaline aqueous solution, and then Ag nanoparticles were anchored to the surface of EG-PDA nanoparticles by the reduction of Ag^+^ by ascorbic acid. The obtained EG-PDA-Ag nanoparticles were subsequently incorporated into a vinyl-methyl-silicone rubber (VMQ) matrix to generate composites with high thermal conductivity. The PDA surface modification effectively reduced the interfacial thermal resistance between EG and VMQ substrate, while the silver nanoparticles effectively established a three-dimensional thermal conductivity network. 10 wt % of EG-PDA-Ag/VMQ composites had a thermal conductivity as high as 1.15 W/mK. This value is six times higher than that of pure VMQ (0.18 W/mK). In addition, the EG-PDA-Ag/VMQ composites exhibit excellent electromechanical and mechanical properties. In conclusion, the proposed method is promising for the future preparation of high thermal conductivity dielectric materials.

## Highlights

- Prepare high thermal conductivity composites to solve the problem of heat dissipation of electrical packaging and components. The 3D interconnection network heat path is effectively provided by expanded graphite.
- Expanded graphite-polydopamine-silver (EG-PDA-Ag) hybrid nanoparticles were synthesized using a simple and environmentally friendly method. The presence of PDA endows the material with excellent insulation and dielectric properties.
- EG was first modified by PDA with a method inspired by mussels, and then successfully assembled by Ag^+^ reduction to anchor silver nanoparticles on the surface of EG-PDA.
- The synergistic effect of Ag and EG leads to the high thermal conductivity of the 10 wt % EG-PDA-Ag/VMQ composites reaching 1.15 W/mK. This value is 6.389 times higher than pure VMQ (0.18 W/mK).
- The EG-PDA-Ag/VMQ composites show excellent electromechanical and mechanical properties.

## 1 Introduction

With the increasing performance demands of modern electronic devices and power batteries, thermal management technologies have become a crucial factor restricting advancements in these fields. The need for thermal interface materials (TIMs) is growing to improve the reliability and lifespan of both electronic devices and power batteries.^1,2^ Polymer-based composites, known for their excellent properties such as chemical resistance, low density, and ease of processing, have become strong candidates in the field of thermal management.^3^ A common approach to enhancing the thermal conductivity of these composites is by incorporating thermally conductive fillers into the polymer matrix.

Expanded graphite (EG), with its exceptionally high in-plane thermal conductivity (132 W·m^–1^·K^–1^), is widely used in the fabrication of TIMs.^4^ However, the high electrical conductivity of EG (10^6^ S/cm) poses significant limitations for its use in thermal management of electronic devices.^5^ Consequently, researchers are increasingly focused on how to enhance the thermal conductivity of these materials while simultaneously suppressing their electrical conductivity, optimizing their performance in thermal management composites. Deng et al.^6^ employed a ball milling process to pretreat expanded graphite (EG), followed by its incorporation into a polyvinylidene fluoride (PVDF) matrix using conventional melt processing, resulting in the formation of a continuous thermal conductivity network. The resulting PVDF/EG composites achieved a thermal conductivity of 0.90 W·m^–1^·K^–1^ (15 wt %).

A crucial point is that most inorganic fillers are chemically inert, leading to weak interfacial interactions with the polymer matrix. This results in poor dispersion of the fillers within the matrix, causing them to easily aggregate and form clusters, thereby increasing interfacial thermal resistance.^7−11^ Thus, coating the filler surface with a nanoscale layer is considered an effective surface modification strategy to enhance interfacial bonding between the fillers and the polymer matrix.^12−18^ Liu et al.^14^ functionalized Al_2_O_3_ by depositing hydroxyl groups and grafting with methyl vinyl-dimethoxysilane, creating Al_2_O_3_@Si. This surface-modified filler was incorporated into modified Poly(phenylene oxide) composites via solution blending and hot pressing. The strong covalent bonds formed between Al_2_O_3_@Si and modified Poly(phenylene oxide) (MPPO) significantly reduced interfacial thermal resistance, resulting in a composite with improved thermal conductivity (1.49 W·m^–1^·K^–1^ at 70% filler loading). Chen et al.^17^ modified h-BN nanoparticles with γ-Amino propyl triethoxysilane (APTES) and incorporated them into a natural rubber matrix to form APTES-hBN/NE composites. The thermal conductivity of the APTES-modified h-BN/NE composite reached 0.209 W·m^–1^·K^–1^, a 27.9% increase over pure natural rubber, compared to only 19.7% improvement for unmodified h-BN composites.

Another important aspect of filler surface modification is the construction of effective thermal conduction paths or networks within the substrate. Fillers are typically randomly dispersed within the polymer matrix, resulting in discontinuous thermal pathways, which increases interfacial thermal resistance and limits overall thermal conductivity. By arranging high-thermal-conductivity fillers in a more orderly fashion or forming a three-dimensional interconnected thermal network. This reduces thermal resistance and significantly enhances the material’s thermal conductivity. This approach is widely applied in polymer composites, electronic packaging materials, and thermal management systems for batteries.^19−21^ Bao et al.^18^ developed 3D sulfonamide-modified expanded graphite with interconnected filler networks by mixing expanded graphite with sulfanilamide aniline, followed by freeze-drying. The EG-SA/EP composites were then fabricated using prefilling and hot-pressing. The modified 3D structure significantly enhanced thermal conductivity, achieving 98 W/m·K with 70 wt % filler, nearly 400 times higher than pure EP. In addition to this, silver nanoparticles have a positive impact on the formation of a continuous thermally conductive network or pathway in the matrix, which reduces the resistance to heat transfer and thus improves the overall thermal conductivity of the material.^22,23^ Dong et al.^16^ developed polyimide/boron nitride (BN) nanosheets/silver nanowire composites by dispersing boron nitride nanosheets in a polyimide matrix through freeze-drying, followed by hot-pressing. The silver nanowires acted as ″thermal bridges,″ significantly enhancing thermal conductivity. At 20 wt % BNNS-AgNW, the composite achieved an in-plane thermal conductivity of 4.75 W/m·K, a 324% increase. This demonstrates the critical role of silver nanowires in improving thermal conductivity in polyimide-based composites.

Polydopamine (PDA) exhibits significant advantages over traditional silane coupling agents, such as KH550 and KH560, in enhancing interfacial bonding capabilities. First, PDA contains abundant functional groups, including catechol and amine groups, enabling it to form both chemical bonds and physical interactions with a wide range of substrates, demonstrating superior versatility compared to silane agents. Second, PDA offers enhanced interfacial strength through its multifaceted binding mechanisms.^24^ Additionally, the deposition process of PDA is simple and environmentally friendly, requiring only a mildly alkaline aqueous solution under ambient conditions.^25^ Moreover, PDA possesses excellent biocompatibility and functionalization potential, making it particularly suitable for biomedical applications and complex material systems.^26^ These features highlight PDA’s unique combination of multifunctionality, universality, and eco-friendliness, positioning it as an ideal candidate for improving interfacial performance in diverse applications.

In this study, expanded graphite (EG) was selected as the filler and incorporated into methyl vinyl silicone rubber (VMQ) to fabricate thermally conductive composites. EG was chosen for its high thermal conductivity, high porosity, low density, and cost-effectiveness.^27^ Subsequently, silver nanoparticles were deposited onto the PDA-coated EG via ascorbic acid reduction of silver ions, forming EG-PDA-Ag. Finally, EG/VMQ, EG-Ag/VMQ, and EG-PDA-Ag/VMQ composites were prepared by hot pressing. A comparative study was conducted to evaluate the effects of the PDA nanolayer and silver nanoparticles on the thermal conductivity, dielectric properties, insulation performance, and mechanical properties of the composites.

## 2 Experimental Section

### 2.1 Materials

Expanded graphite (SIGRATHERM GFG) was obtained from SLG Carbon SE., Germany. Tris(hydroxymethyl)aminomethane, polyvinylpyrrolidone (PVP)(Average Molecular Wt.40000), Ascorbic Acid(>99.0%)were purchased from TCI Deutschland GmbH. In addition, polydimethylsiloxane silicone rubber (VMQ) (LUMISIL® LR 7601/50 A/B) were obtained through Wacker Chemie AG, Germany. Dopamine hydrochloride (99%) was purchased from Thermo Fisher Scientific Inc., Germany. AgNO_3_ (99%) was purchased from Feinchemikalien and Forschungsbedarf GmbH, Germany.

### 2.2 Preparation of EG-PDA-Ag

Polydopamine (PDA) nanolayers were successfully coated onto the surface of expanded graphite (EG) as a surface modification step, followed by the attachment of silver (Ag) nanoparticles. The PDA deposition process is described in greater detail in a previous study.^28^ The process began by dissolving 0.24 g of dopamine hydrochloride in 64 mL of deionized water (3.75 g/L). Tris was then added to the solution to create a buffer, adjusting the pH to 8.5. Subsequently, 1.4 g of EG was dispersed into the solution and magnetically stirred at 25 °C for 24 h. The reaction suspension was then filtered and thoroughly washed with deionized water, yielding dopamine-modified expanded graphite (EG-PDA). The EG-PDA was vacuum-dried at 100 °C for 24 h to obtain the final modified filler.

**Figure 1:**
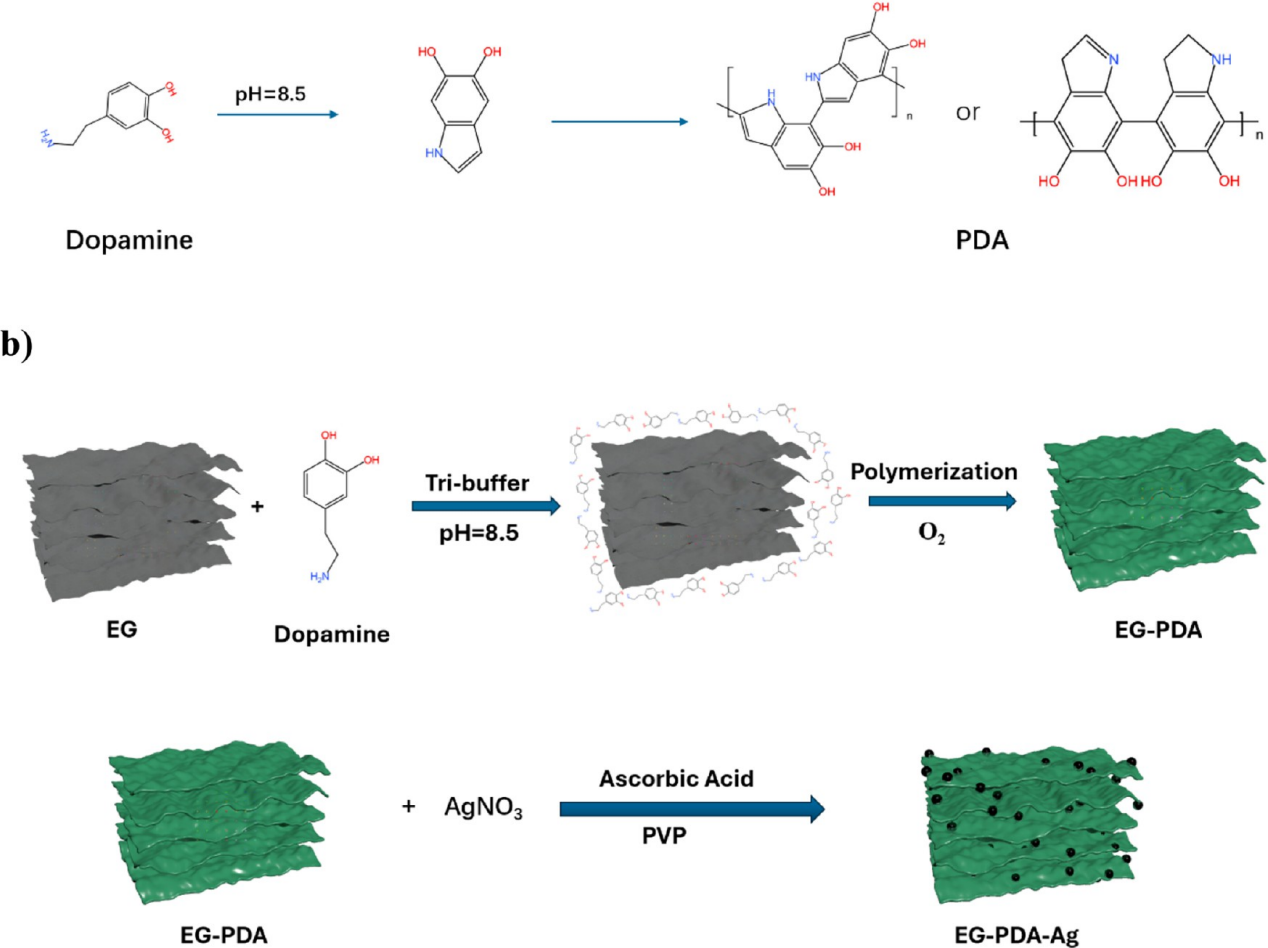
Schematic of (a) mechanism of dopamine oxidative self-polymerization and (b) synthesis of EG-PDA-Ag.

For Ag nanoparticle attachment,^29^ 0.85 g of AgNO_3_ was dissolved in 50 mL of deionized water, followed by the addition of 1 g of EG-PDA and 0.85 g of polyvinylpyrrolidone (PVP). PVP functions as a binder, facilitating the adhesion of the reduced silver nanoparticles to the surface of the filler.^30^ The mixture was stirred for 20 min, after which 90 mL of a 2 wt % ascorbic acid solution (prepared by dissolving 1.8 g of ascorbic acid in 90 mL deionized water) was slowly added while stirring. The reaction was continued for an additional 2 h. The final suspension was filtered, washed with deionized water, and vacuum-dried at 80 °C for 12 h, resulting in Ag nanoparticle-modified expanded graphite (EG-PDA-Ag). The schematic of the preparation process of EG-PDA-Ag and the mechanism of oxidative self-polymerization of PDA are shown Figure 1 below.

### 2.3 Preparation of VMQ Composites

Various mass fractions (0, 2.5, 5, 7.5, and 10 wt %) of EG, EG-Ag or EG-PDA-Ag were blended with preconfigured VMQ at ambient temperature to form a uniformly dispersed suspension. This suspension was subsequently subjected to hot-pressing using a high-temperature hot-pressing machine at 150 °C and 120 MPa for 15 min, followed by cold-pressing at 120 MPa for 5 min to yield the final thermally conductive and insulating silicone rubber composite. The resulting thermally conductive and insulating silicone rubber materials, utilizing EG, EG-Ag, and EG-PDA-Ag as fillers, were designated as EG/VMQ, EG-Ag/VMQ, and EG-PDA-Ag/VMQ, respectively. The blending procedure involving different fillers and the VMQ matrix is illustrated in the Figure S1.

### 2.4 Characterization Methods

Fourier transform infrared (FTIR) spectroscopy was conducted in the range of 4000–400 cm^–1^ using a Thermo Nicolet 380 spectrometer to investigate the presence of intermolecular chemical bonds.^31^ Morphological and elemental analyses were performed by scanning electron microscopy (SEM) and energy-dispersive X-ray spectrometry (EDX) using a Hitachi S-4800 instrument. High-resolution transmission electron microscopy (HR-TEM) was carried out using a field-emission TEM (TALOS F200X).^32^ The elemental composition of the samples was characterized by X-ray photoelectron spectroscopy (XPS) on a Thermo Electron Escalab 250Xi instrument.^33^ The crystal structures of EG, EG-Ag, and EG-PDA-Ag were analyzed by X-ray diffraction (XRD) using a Rigaku Ultima IV diffractometer with Cu Kβ radiation (λ = 0.154 nm) under the following conditions: scanning speed of 10°/min, 2θ range of 5°–35°, 40 kV generator voltage, and 40 mA tube current.^34^ The thermal stability of the composites was evaluated by thermogravimetric analysis (TGA) using a STA409 PC/PG instrument under a nitrogen atmosphere with a heating rate of 10 °C min^–1^.^35^ The thermal conductivity of the samples was measured with a Hot Wire thermal coefficient detector (Xiatech TC3000E) at a testing voltage of 2.5 V, with five individual measurements taken for each sample. Tensile properties were assessed following the ISO 527–1 standard, using a Zwick Roell ProLine universal tensile testing machine at room temperature, with a strain rate of 10 mm/min to generate stress–strain curves. Dielectric properties and volume resistivity of the VMQ composites were measured in the frequency range of 100–10^7^ Hz at room temperature using a Concept 40 Alpha-A broadband dielectric spectrometer (Novocontrol GmbH,Germany).

## 3 Results and Discussion

### 3.1 Microstructure and Characterization of EG, EG-Ag, and EG-PDA-Ag

**Figure 2:**
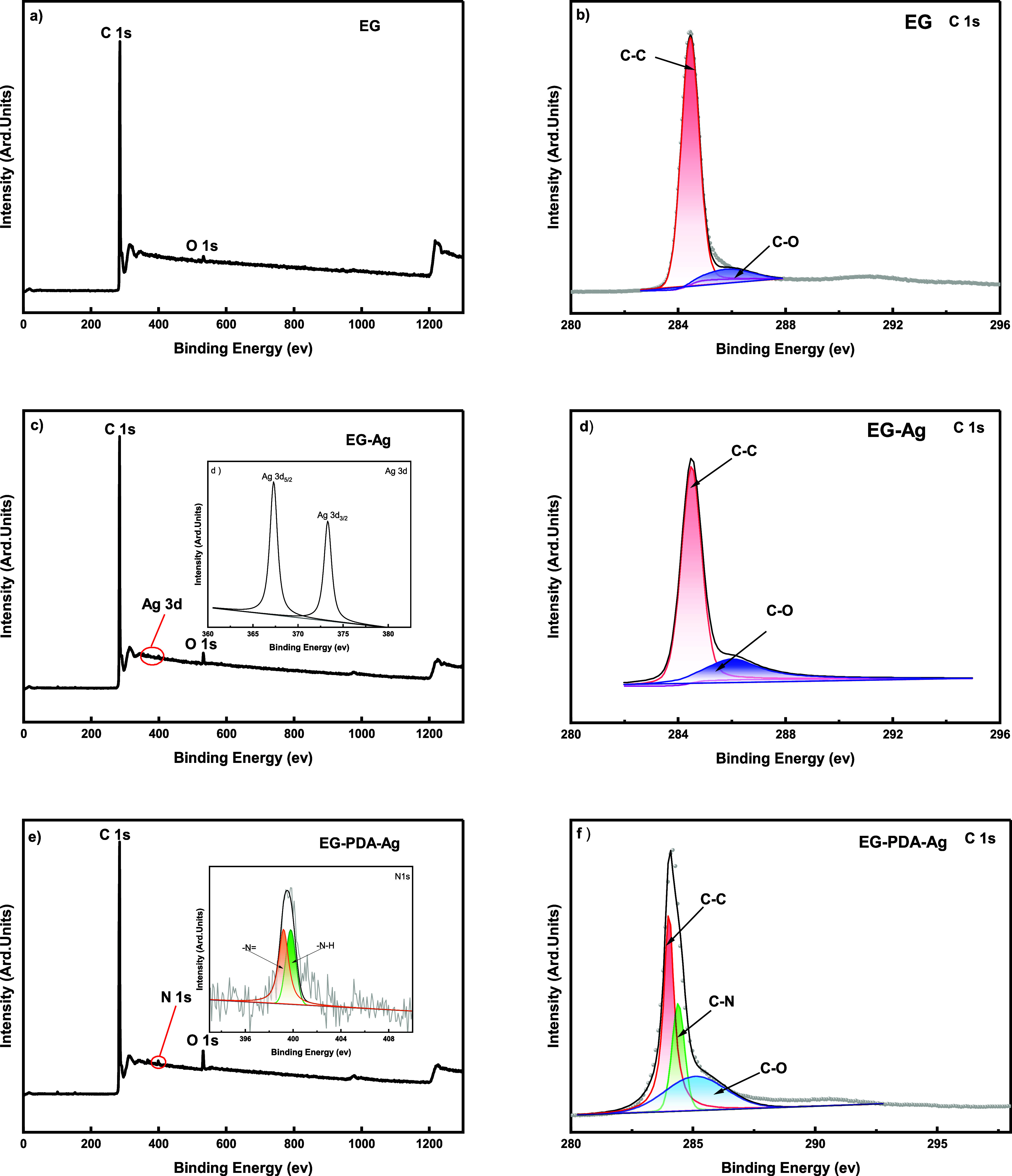
XPS spectra of EG and EG-Ag and EG-PDA-Ag: (a) General spectrum of EG, (c) general spectrum of EG-Ag with the spectrum of Ag 3d, (e) general spectrum of EG-PDA-Ag with the spectrum of N 1s; (b) C 1s spectrum of EG, (d) C 1s spectrum of EG-Ag, (f) C 1s spectrum of EG-PDA-Ag.

XPS analysis results show the surface element composition of original EG, EG-Ag and EG-PDA-Ag. Figure 2 illustrates the XPS analysis of EG, EG-Ag, and EG-PDA-Ag fillers. All samples exhibited C 1s and O 1s peaks, confirming the presence of carbon and oxygen. Ag 3d peaks were observed in EG-Ag and EG-PDA-Ag, with binding energies of 368.2 and 374.2 eV, corresponding to metallic silver (Ag^0^), and the absence of Ag^+^ peaks indicated the successful reduction of Ag ions to Ag nanoparticles on the filler surface. The C 1s spectra for EG and EG-Ag were identical,

showing only C–C and C- O bonds, confirming that the silver modification did not alter the chemical structure of EG. In EG-PDA-Ag, a new N 1s peak at ∼ 400 eV was detected, corresponding to = N and N–H bonds in the polydopamine (PDA) layer, which was formed through dopamine’s oxidative self-polymerization under weak alkaline conditions. The presence of a C–N bond at 284.8 eV in the C 1s spectrum further verified successful surface modification of EG by PDA. These results collectively confirm the effective deposition of both PDA and Ag nanoparticles without altering the core structure of the expanded graphite.

Figure S2(a) presents the TGA curves of EG, EG-Ag, and EG-PDA-Ag under nitrogen, showing distinct weight loss profiles at 750 °C. The weight losses were found to be 0.02% for EG, 1.37% for EG-Ag, and 3.03% for EG-PDA-Ag. Since EG alone exhibited negligible weight loss, it was attributed to the release of trapped oxygen in the graphite pores. For EG-Ag, the additional weight loss of 1.35% can be attributed to the oxidation and subsequent thermal decomposition of silver nanoparticles. In EG-PDA-Ag, the total weight loss of 3.01% suggests the combined effects of silver nanoparticle oxidation and PDA decomposition. Given the known decomposition rate of PDA (50.2% at 750 °C), the proportion of PDA in the EG-PDA-Ag sample is estimated at approximately 2.66%.^36^ This indicates that silver accounts for the remaining ∼ 0.35%, reflecting its oxidation and reduction during heating. These results highlight the significant contributions of PDA and silver to the overall thermal stability and behavior of the composites. FTIR analysis was performed to examine the chemical structures of EG, EG-Ag, and EG-PDA-Ag, as illustrated in Figure S2(b). The spectra of all three materials display characteristic absorption peaks at 1086 cm^–1^, 2923 cm^–1^, and 3435 cm^–1^, corresponding to C–OH stretching, C–H stretching, and O–H stretching vibrations, respectively. Notably, the FTIR spectrum of EG-Ag does not exhibit any significant new absorption peaks compared to EG, suggesting that the silver is present as metallic Ag, without forming chemical bonds with carbon in the graphite structure. In contrast, the spectrum of EG-PDA-Ag reveals new absorption peaks at 1246 cm^–1^ and 1782 cm^–1^, which are attributed to the C–N stretching and N–H bending vibrations in polydopamine (PDA), respectively.^37^ These observations confirm the successful encapsulation of PDA on the surface of EG, further modifying its chemical structure.

X-ray diffraction (XRD) analysis was conducted to investigate the crystal structure and phase composition of EG, EG-Ag, and EG-PDA-Ag, as shown in Figure S3. The XRD patterns of EG-Ag and EG-PDA-Ag exhibit two additional peaks at 38.1° and 44.3°, corresponding to the (111) and (200) planes of face-centered cubic (FCC) silver, consistent with the standard JCPDS card (No. 04–0783), confirming the presence of a pure silver phase. Other than these Ag-related peaks, the diffraction patterns of EG-Ag and EG-PDA-Ag are consistent with pure EG, indicating that the dopamine modification does not affect the crystal structure of EG, and the PDA layer is amorphous. Moreover, the reduced intensity of the Ag (111) and (200) peaks in EG-PDA-Ag suggests that PDA effectively reduces the agglomeration of Ag nanoparticles, resulting in a more uniform distribution on the EG surface.

**Figure 3:**
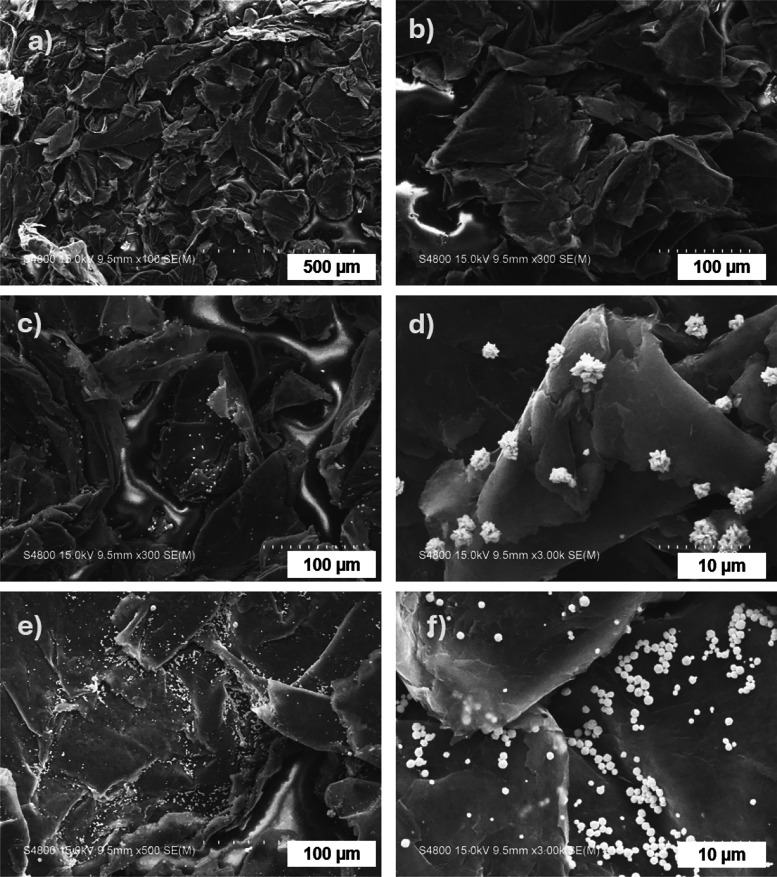
SEM image of the surface of fillers of EG (a) at 500 and (b) at 100 μm; EG-Ag (c) at 100 and (d) at 10 μm; EG-PDA-Ag (e) at 100 and (f) at 10 μm scales.

To investigate the effects of PDA and Ag surface modification on filler dispersion and interfacial bonding, SEM images of EG, EG-Ag, and EG-PDA-Ag were obtained **(**Figure 3**).** The EG samples **(**Figure 3**a, b)** exhibit a typical lamellar structure with visible interlayer porosity and well-aligned layers, although some cracks and holes likely resulted from the expansion process. In contrast, SEM images of EG-Ag **(**Figure 3**c, d)** reveal a large number of Ag nanoparticles attached to the EG surface, but they are unevenly distributed and tend to aggregate due to poor interfacial compatibility and the high surface energy of Ag. After PDA coating **(**Figure 3**e, f)**, the silver nanoparticles are more uniformly dispersed without significant agglomeration. The PDA layer effectively reduces surface energy, provides binding sites via its functional groups, and forms a protective layer, preventing direct Ag nanoparticle contact and agglomeration. This improved dispersion of Ag nanoparticles enhances the stability and interfacial bonding of the composite.

**Figure 4:**
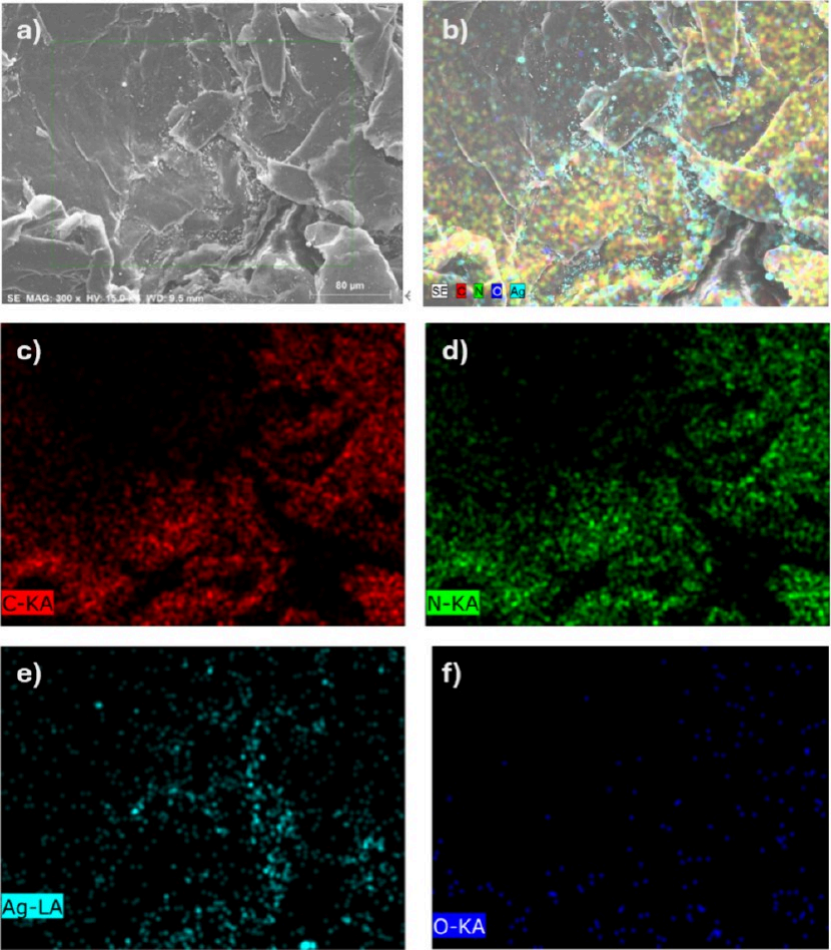
EDX mapping analysis on EG-PDA-Ag; (a) SEM image and (b) overall mapping elements on the same spot, corresponding to carbon (c), nitrogen (d), silver (e), and oxygen (f) mapping.

The spatial distribution of elements C, N, O, and Ag in the composites is illustrated in the elemental mapping in Figure 4. Carbon (C) is uniformly dispersed throughout the substrate, confirming the even distribution of expanded graphite. Oxygen (O) is also evenly distributed, indicating successful coverage of the PDA coating on the composite surface. The uniform distribution of silver (Ag) nanoparticles further confirms their successful deposition on the EG-PDA surface, consistent with the intended dispersion. This result verifies the effective integration of both PDA and Ag into the composite material.

**Figure 5:**
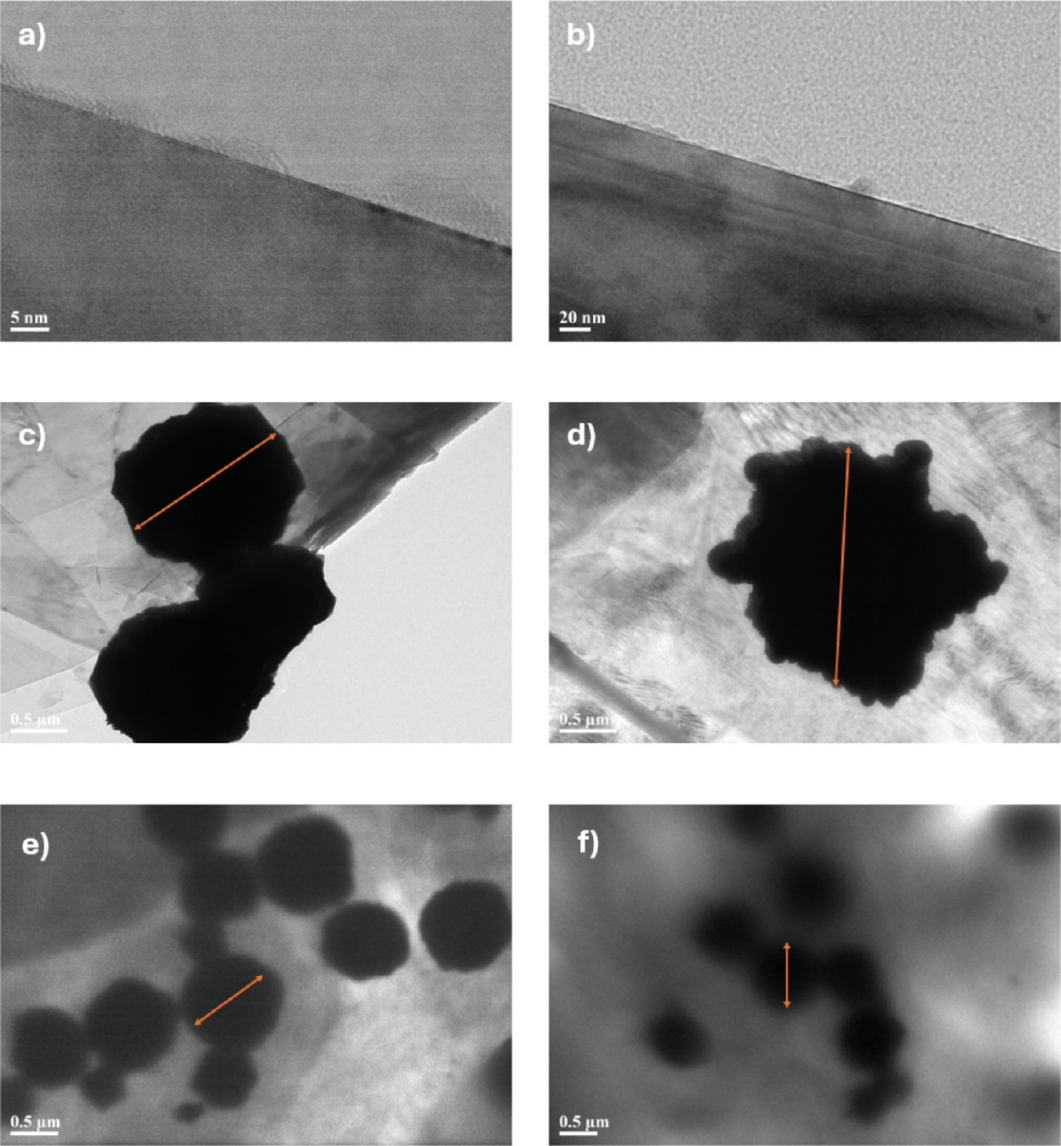
High-resolution transmission electron microscope diagrams of (a, b) EG; (c, d) EG-Ag; (e, f) EG-PDA-Ag.

High-resolution transmission electron microscopy (HR-TEM) was used to analyze the microstructure of EG, EG-Ag, and EG-PDA-Ag, as shown in Figure 5. The pure expanded graphite (EG) sample, displayed in Figures 5(a) and 5(b), reveals a typical layered structure with visible spacing between the graphite layers and no evident impurities, indicating high purity. In contrast, the HR-TEM images of EG-Ag, Figure 5**(c, d)**, show clusters of unevenly distributed silver nanoparticles (4–6 μm in size) tightly attached to the graphite surface, confirming successful Ag loading via chemical reduction. However, poor interfacial compatibility led to agglomeration of Ag nanoparticles. In Figure 5**(e, f)**, the EG-PDA-Ag sample exhibits a more uniform distribution of smaller silver nanoparticles with minimal agglomeration. The presence of a thin polydopamine layer between the Ag nanoparticles and the graphite surface improves nanoparticle dispersion and enhances adhesion, thereby improving the material’s thermal conductivity.

### 3.2 Microstructure of VMQ Composites Filled with EG, EG-Ag, and EG-Ag-PDA

**Figure 6:**
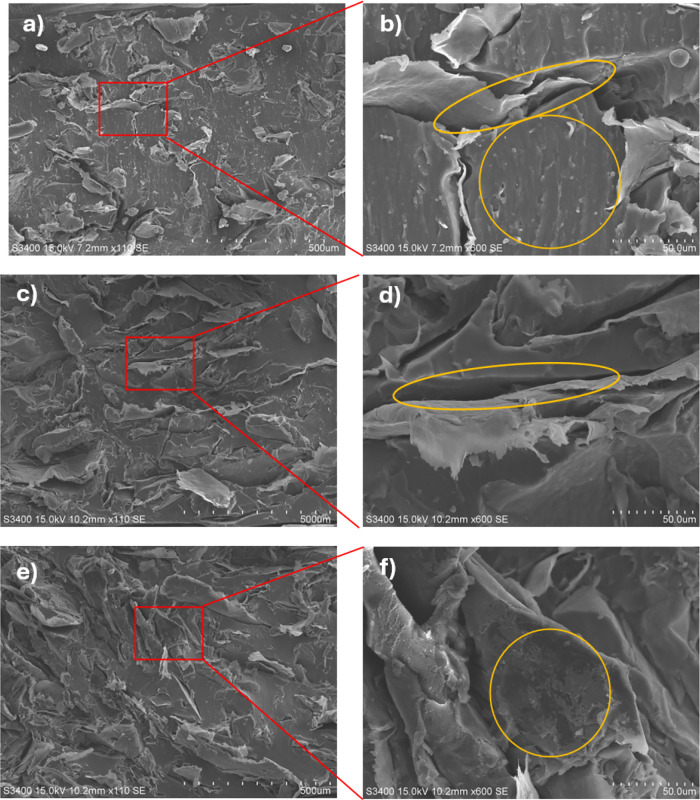
(a, b) SEM image of the fracture surface of 10 wt % EG/VMQ composite; (c, d) SEM image of the fracture surface of 10 wt % EG-Ag/VMQ composite; (e, f) SEM image of the fracture surface of 10 wt % EG-PDA-Ag/VMQ composite. Hydrogen bonds, with the VMQ substrate, facilitating better adhesion and effectively bridging neighboring EG particles. This results in a synergistic effect that significantly improves the thermal conductivity of the composite.

To investigate the effects of polydopamine (PDA) and silver (Ag) modifications on the dispersion and interfacial bonding of fillers in EG/VMQ composites, scanning electron microscopy (SEM) images of the fracture surfaces were analyzed for samples with 10 wt % loading of EG/VMQ, EG-Ag/VMQ, and EG-PDA-Ag/VMQ, as shown in Figure 6**(a,b)**. The EG particles in the EG/VMQ composite exhibit a relatively uniform distribution, yet notable voids and interfacial cracks indicate inadequate bonding due to the physical adsorption and van der Waals forces between the inert EG surface and the VMQ substrate. In contrast in Figure 6**(c,d)**, the EG-Ag/VMQ composite shows improved filler distribution, with a little bit smoother interface and reduced cracks than EG/VMQ, this is due to the high specific surface area of silver nanoparticles, which enhances physical adsorption and increases contact area between the fillers and the substrate. Finally in Figure 6**(e,f)**, the EG-PDA-Ag composite demonstrates good dispersion and a tight bond with the VMQ matrix. This enhancement is attributed to the PDA coating, which promotes stronger chemical interactions, including covalent and

### 3.3 Thermal Conductivity of VMQ Composites

**Figure 7:**
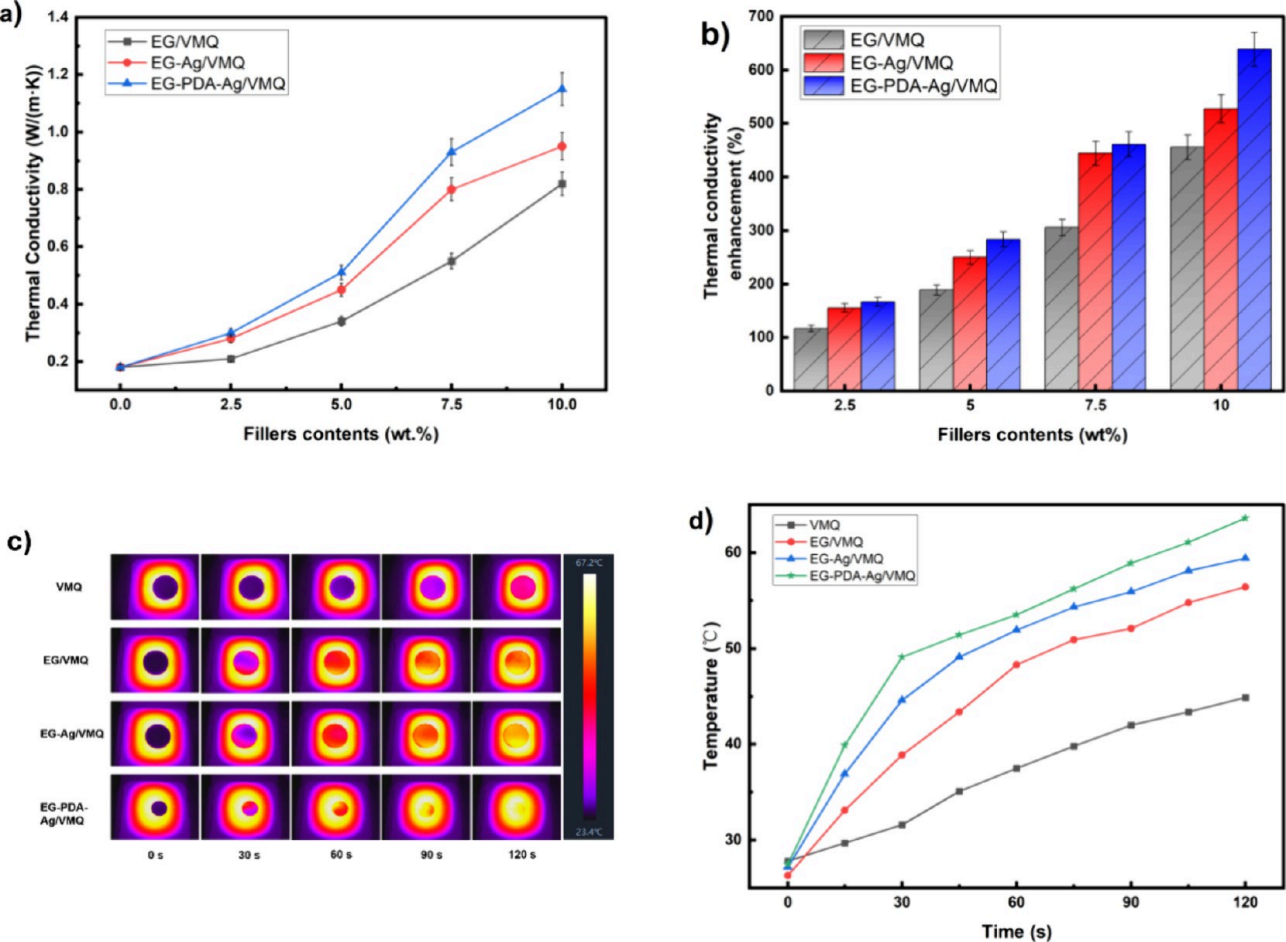
(a) Thermal conductivity curves of EG/VMQ and EG-Ag/VMQ and EG-PDA-Ag/VMQ with different filler loadings. (b) Percentage increase in thermal conductivity for different filler loadings. (c) Infrared thermographic images of VMQ, EG/VMQ, EG-Ag/VMQ, and EG-PDA-Ag/VMQ at 10% filler loading during the heating process. (d) Surface temperature profiles of VMQ, EG/VMQ, EG-Ag/VMQ, and EG-PDA-Ag/VMQ during heating vs time with 10% different fillers.

Figure 7(a) demonstrates that the thermal conductivity of the composites—EG/VMQ, EG-Ag/VMQ, and EG-PDA-Ag/VMQ—exhibits an increasing trend with higher filler loading. In Figure 7, EG-PDA/VMQ was not added in this series of study curves, mainly because the focus of this study was to highlight the effect of the presence of PDA on the growth and distribution of Ag nanoparticles and how it further affects the thermal conductivity. According to thermal path theory, at low filler loadings, the thermally conductive fillers are dispersed and do not make contact with each other, resulting in a ″island″ structure that limits the enhancement of thermal conductivity. As the filler loading increases, the fillers come into contact and establish thermal conductive pathways or networks, thereby enhancing the thermal conductivity of the composites more effectively.

To quantify the effects of Ag nanoparticles and PDA modification on the thermal conductivity enhancement of thermally conductive insulating silicone rubber (VMQ), the concept of Thermal Conductivity Enhancement (TCE)^38^ is introduced, defined by the equation:
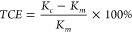
1Where K_c_ and K_m_ represent the thermal conductivities of the thermally conductive insulating silicone rubber composite and the pure VMQ matrix, respectively. The thermal conductivity of the pure VMQ matrix at room temperature is known to be 0.18 W/m·K.

Using eq 1, Figure 7(b) shows the percentage increase in thermal conductivity of the composites with varying fillers compared to pure VMQ at different loading levels. The results reveal that thermal conductivity improvement correlates positively with filler loading for the same filler type, consistent with the trends in Figure 7(a). While the differences in enhancements among EG/VMQ, EG-Ag/VMQ, and EG-PDA-Ag/VMQ are minimal at low loadings, they become more pronounced at higher loadings. Notably, at 10 wt % filler loading, the thermal conductivity of EG-PDA-Ag/VMQ reaches 1.15 W·m^–1^·K^–1^, representing 638.9% of that of the pure VMQ matrix and a 38.4% increase compared to EG-Ag/VMQ at the same loading. These findings indicate that the PDA surface modification significantly enhances the thermal conductivity of thermally conductive insulating silicone rubber materials.

The observed enhancement in thermal conductivity of EG-PDA-Ag/VMQ composites can be attributed to several key factors. Initially, pure VMQ conducts heat primarily through phonon vibrations generated by its atomic and polymeric structures. At low filler loadings of EG-PDA-Ag, an effective thermal conductivity network is not formed due to insufficient contact between the fillers, resulting in only slight improvements in thermal conductivity. However, as the loading of EG-PDA-Ag increases, an effective thermal pathway develops between the fillers, significantly enhancing the composite’s thermal conductivity. This improvement can be linked to the intrinsic high thermal conductivity of silver nanoparticles, as well as the enhanced interfacial bonding between the fillers and the VMQ matrix due to PDA incorporation, which reduces interfacial thermal resistance compared to both EG/VMQ and EG-Ag/VMQ systems.

Moreover, the homogeneous distribution of EG-PDA-Ag within the VMQ matrix—free from large agglomerations—further promotes effective thermal conduction. The excellent compatibility between the inorganic nanoparticles and the organic matrix, facilitated by PDA’s strong adhesion properties, enables the formation of a continuous thermal conduction pathway, minimizing heat scattering and loss. Additionally, the presence of silver nanoparticles allows for effective bridging between adjacent EG particles, leading to a synergistic effect that enhances the overall thermal conductivity of the composite.

To intuitively compare the thermal conductivity of VMQ, EG/VMQ, EG-Ag/VMQ, and EG-PDA-Ag/VMQ, composites with a 10 wt % filler loading were subjected to a uniform heat source, and their surface temperatures were recorded every 15 s using a hand-held infrared thermal imager. The resulting temperature change graph is presented in Figure 7(c). Initially, the materials displayed similar colors, indicating nearly identical starting temperatures. However, over time, the composites showed a more rapid change in color compared to pure VMQ, with the thermographic images of the composites becoming lighter, signifying enhanced thermal conductivity and increased heat transfer rates due to the fillers. Notably, after 120 s of heating, the EG-PDA-Ag/VMQ sample exhibited the lightest color, reflecting a faster rise in surface temperature and confirming its superior thermal conductivity.

Figure 7(d) illustrates the surface temperatures of the various materials at different time intervals. The results reveal that the three composites consistently maintained higher temperatures than pure VMQ, underscoring the significant enhancement in thermal conductivity conferred by the thermally conductive fillers. At any given time, the surface temperature of EG-PDA-Ag/VMQ exceeded that of EG-Ag/VMQ, which, in turn, was higher than that of EG/VMQ. This hierarchy indicates that EG-PDA-Ag demonstrates the highest thermal efficiency, followed by EG-Ag and EG. These findings are consistent with previously measured thermal conductivity data, further confirming the positive impact of silver nanoparticles on the thermal performance of EG and highlighting the superior thermal conductivity of the EG-PDA-Ag/VMQ composite with PDA surface modification.

### 3.4 Thermal Conductivity Mechanism in VMQ Composites

The degree of interfacial bonding significantly influences the thermal conductivity of materials. The thermal conductivity differences among the composites EG/VMQ, EG-Ag/VMQ, and EG-PDA-Ag/VMQ become more pronounced with increasing filler loading. At higher loadings, a continuous thermal network forms, enhancing the interfacial bonding between EG-PDA-Ag and VMQ, which facilitates more efficient phonon transport and reduces interfacial thermal resistance. This results in a greater disparity in thermal conductivity among the composites. Conversely, at lower filler loadings, where the structure resembles an isolated ″island″ configuration, the contributions to thermal conductivity from EG, EG-Ag, and EG-PDA-Ag are relatively similar, leading to less pronounced differences in thermal performance. Additionally, PDA modification improves the dispersibility of the EG filler, minimizing agglomeration tendencies. The incorporation of silver nanoparticles allows for effective bridging between adjacent EG particles, generating a synergistic effect that enhances thermal conductivity. Consequently, even at equivalent filler loadings, the thermal conductivity of EG-PDA-Ag/VMQ remains significantly higher than that of EG-Ag/VMQ due to the improved dispersion and reduced agglomeration of the fillers.

The influence of thermal resistance on thermal conductivity is commonly elucidated using the effective medium theory (EMT) or the Foygel model. The former assumes that the fillers are entirely encapsulated by the polymer matrix, making it suitable for calculating the filler/polymer interfacial thermal resistance (*R*_*b*_) in low-filler-content composites. However, for the three types of composites with interconnected filler network structures considered in this study, the filler/filler interfacial thermal resistance (*R*_*c*_) is the dominant factor affecting the overall thermal conduction process within the composites. Therefore, the Foygel model is employed in this work to elucidate *R*_*c*_. The model is described by the following equation:^39,40^
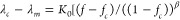
2
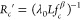
3
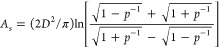
4
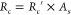
5

In the equation, λ_*m*_ and λ_*c*_ represent the thermal conductivity of the VMQ matrix (0.175 W·m^–1^·K^–1^) and the composite, respectively. *K*_*0*_ is the pre-exponential factor influenced by the filler arrangement, while β is an exponent dependent on the aspect ratio of EG. *D* denotes the thickness of EG, *L* represents its lateral dimension, and *p(L/D)* corresponds to the aspect ratio of EG. The critical loading of EG, *f*_*c*_ is defined *f*_*c*_*=* 0.6*/p* when *p*≫1.42. The parameters used to fit the thermal conductivity of EG/VMQ, EG-Ag/VMQ and EG-PDA-Ag/VMQS composites using the Foygel model^41^ are listed in Table S1. The corresponding fitting curves are presented in Figure S4. As displayed in Table S1, the R_c_ of EG-PDA-Ag/VMQ composites was 4.94 × 10^–7^ m^2^KW^–1^, which was lower than that of EG/VMQ composites (5.22 × 10^–7^ m^2^KW^–1^) and EG-Ag/VMQ composites (5.53 × 10^–7^ m^2^KW^–1^), respectively. This qualitatively explains why the EG-PDA-Ag/VMQ composites have higher K compared with the EG/VMQ composites.

Based on the thermal conductivity and *Rc* analysis of composite materials, the thermal conduction mechanism of composite materials filled with different types of fillers is proposed and demonstrated in Figure S5. It illustrates the thermal conductivity models of EG/VMQ, EG-Ag/VMQ, and EG-PDA-Ag/VMQ, highlighting that EG-PDA-Ag forms thermal pathways or networks more efficiently than EG and EG-Ag at the same filler loading, thereby achieving higher thermal conductivity. As shown in Figure 7(a), the thermal conductivity of EG-PDA-Ag/VMQ at 7.5 wt % loading is comparable to that of EG-Ag/VMQ at 10 wt %, indicating that EG-PDA-Ag can establish effective continuous thermal conductive pathways at lower loadings. The polydopamine layer enhances compatibility with the VMQ matrix, allowing phonons to propagate effectively at the interface with reduced inelastic scattering, resulting in a higher phonon mean free path and lower interfacial thermal resistance. This leads to a significant increase in the thermal conductivity of the composites. The PDA modification enhances thermal conductivity efficiency and improves dispersion uniformity and interfacial bonding. Furthermore, the incorporation of silver nanoparticles facilitates effective bridging between adjacent EG particles, generating a synergistic effect that forms a continuous thermal conduction path at lower loadings. This approach effectively addresses issues related to low mechanical properties, poor molding and processing performance, and high costs associated with excessive filler addition.

**Figure 8:**
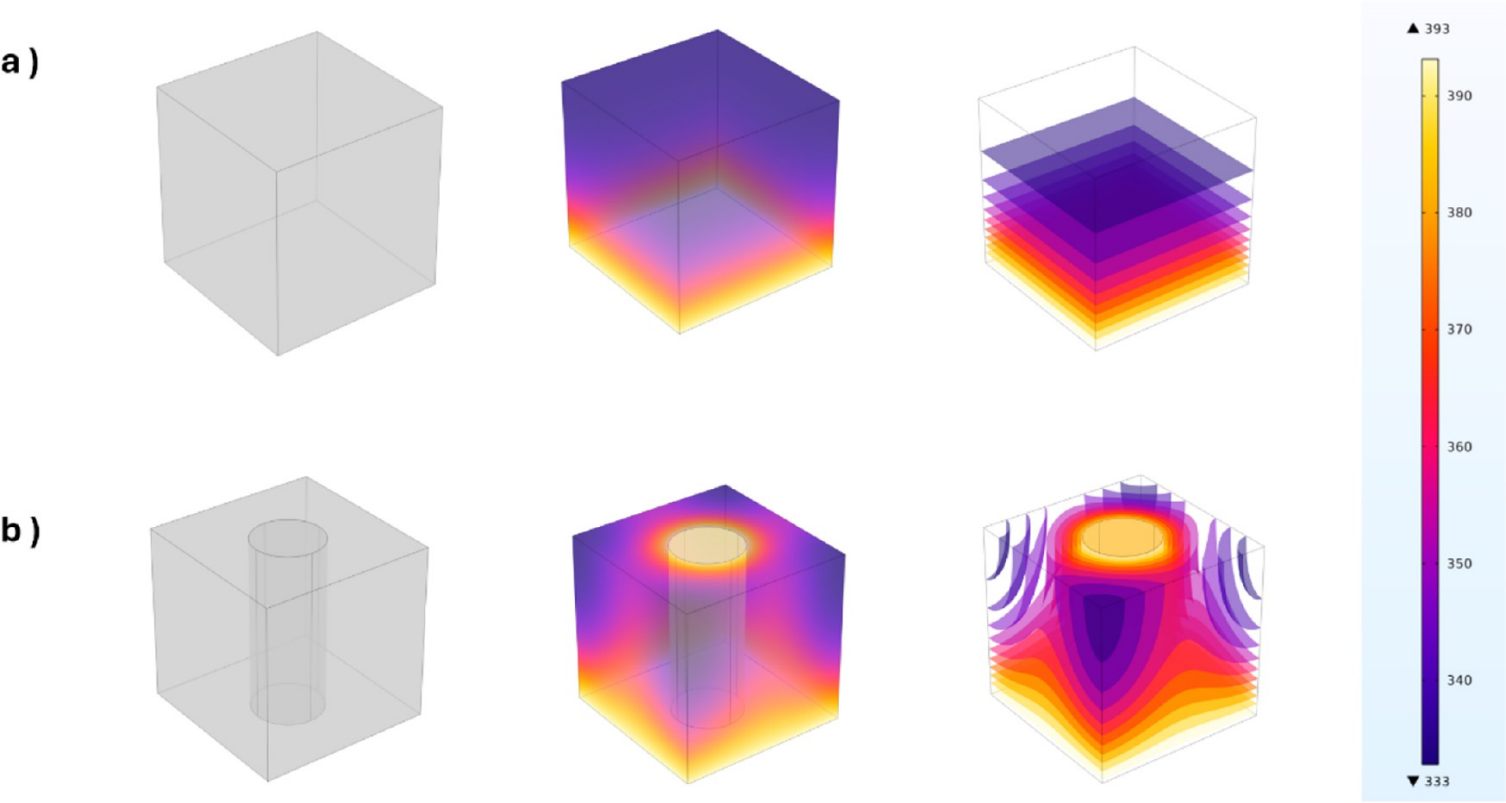
3D pictures of (a) pure VMQ and (b) EG-PDA-Ag/VMQ thermally conductive silicone rubber composites with thermal simulation (COMSOL 6.2).

Figure 8 depicts a simulation of the composites’ thermal conductivity by applying COMSOL software. In this simulation, the transient solid thermal conductivity method was applied to load the heat source from the bottom, raising the temperature from room temperature (293 K) to 393 K. The temperature distribution and isothermal surfaces within the materials were simulated over the same period. As shown in Figure 8(a), the temperature distribution in pure VMQ exhibits a uniform upward trend, with heat steadily transferring from the bottom to the top, as reflected by the smooth isothermal surfaces. In contrast, Figure 8(b) shows that when the heat reaches the EG-PDA-Ag thermal conductivity path, the temperature around the filler rises sharply due to its high thermal conductivity, rapidly transferring heat to the top of the material and resulting in a steeper temperature gradient. This demonstrates that EG-PDA-Ag significantly enhances the thermal conductivity of VMQ by forming a three-dimensional interconnected network, confirming the synergistic effects of PDA and Ag nanoparticles in boosting heat transfer efficiency.

To emphasize the superior thermal conductivity of the 10 wt % EG-PDA-Ag/VMQ composites, a comparison was made between the filler content and thermal conductivity with other studies using expanded graphite or modified fillers. As shown in Figure S6, the thermal conductivity of EG-PDA-Ag/VMQ in this study surpasses that of similar composites at comparable filler levels.^6,13,42−45^ For instance, Liu et al.’s capric acid (CA)-stearic acid (SA)/9 wt % EG composite achieved 0.942 W·m^–1^·K^–1^, while Song et al.’s MgCl_2_-6H_2_O/10 wt % EG composite reached only 0.5218 W·m^–1^·K^–1^. These findings highlight the significant improvement in thermal conductivity attributed to the synergistic effects of PDA surface modification and silver nanoparticle incorporation, underscoring the competitive potential of the EG-PDA-Ag/VMQ composites for thermal management applications.

### 3.5 Dielectric Properties of VMQ Composites

**Figure 9:**
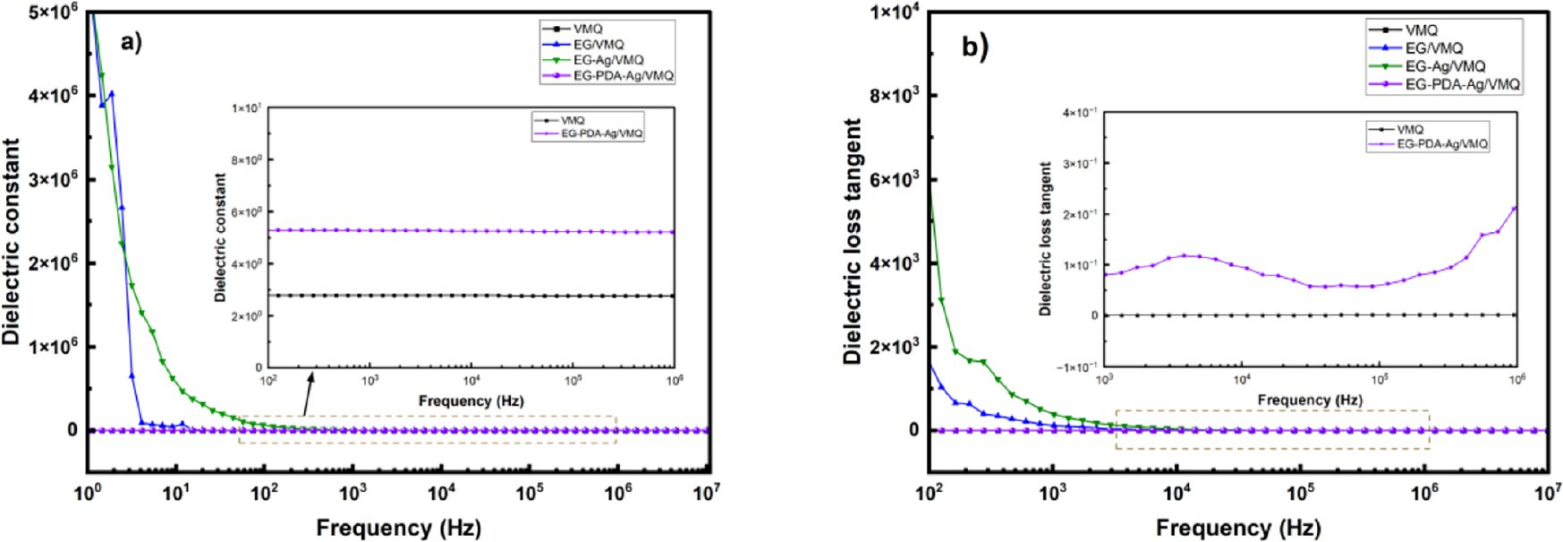
Frequency dependence of (a) dielectric constant (b) dielectric loss tangent of VMQ, EG/VMQ, EG-Ag/VMQ, and EG-PDA-Ag/VMQ.

Figure 9(a) compares the dielectric constant of pure VMQ, EG/VMQ, EG-Ag/VMQ, and EG-PDA-Ag/VMQ composites across varying electric field frequencies. Both pure VMQ and EG-PDA-Ag/VMQ exhibit stable and low dielectric constants, with EG-PDA-Ag/VMQ slightly higher due to Ag nanoparticles enhancing interfacial polarization (Maxwell–Wagner-Sillars effect).^24^ In contrast, the dielectric constants of EG/VMQ and EG-Ag/VMQ decrease with increasing frequency due to polarization relaxation, with EG-Ag/VMQ showing consistently higher values due to the influence of silver nanoparticles.

Similarly, Figure 9(b) shows that the dielectric loss (tan δ) of pure VMQ and EG-PDA-Ag/VMQ remains low and stable across the frequency range, with EG-PDA-Ag/VMQ slightly higher than pure VMQ due to the improved dispersion and stability provided by PDA modification and Ag nanoparticles. In contrast, EG/VMQ and EG-Ag/VMQ exhibit higher tan δ values at low frequencies, decreasing at higher frequencies as the polarization response weakens. The enhanced interfacial polarization and conductivity from Ag nanoparticles in EG-Ag/VMQ further elevate tan δ.

Overall, EG-PDA-Ag/VMQ demonstrates lower and more stable dielectric properties than EG/VMQ or EG-Ag/VMQ, making it highly suitable for use in electronic thermal interface materials (TIMs) where both thermal conductivity and favorable dielectric properties are essential for operational stability and reliability.

### 3.6 Electrical Properties of VMQ Composites

**Figure 10:**
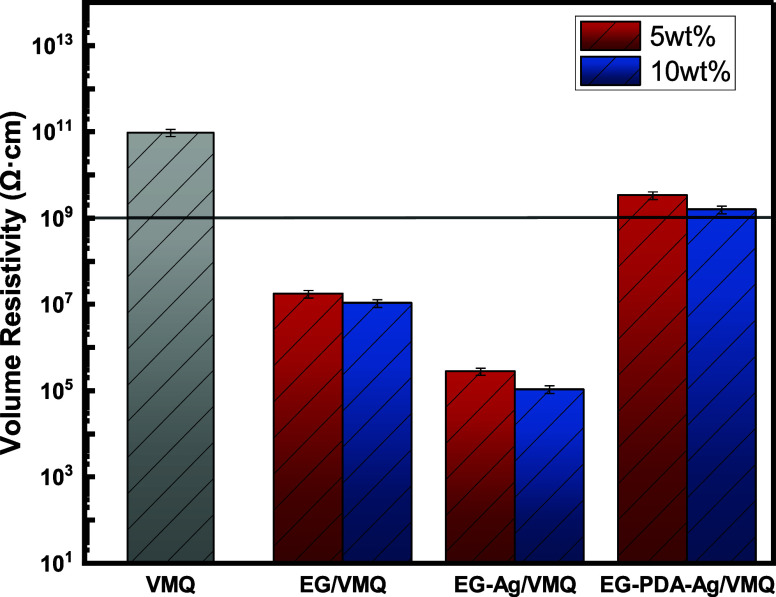
Volume resistivity of VMQ,EG/VMQ and EG-Ag/VMQ and EG-PDA-Ag/VMQ under different filler loadings.

The volume resistivity of VMQ as well as EG/VMQ and EG-Ag/VMQ and EG-PDA-Ag/VMQ with different filler loadings are given in Figure 10. At 50 Hz AC, the volume resistivity of pure VMQ is about 10^11^ Ω·cm, demonstrating excellent electrical insulation properties. However, adding unmodified EG significantly reduces resistivity, with 5 wt % EG/VMQ decreasing to 1.74 × 10^7^ Ω·cm, a drop of 4 orders of magnitude. This is attributed to increased filler loading, which leads to denser distribution of EG particles, more conductive paths, and larger contact areas, facilitating electron transfer. At 10 wt %, resistivity further decreases by 38.8% to 1.06 × 10^7^ Ω·cm. For EG-Ag/VMQ composites, resistivity decreases even more drastically due to the superior conductivity of silver. At 5 and 10 wt %, resistivities of 2.79 × 10^5^ Ω·cm and 1.07 × 10^5^ Ω·cm are observed, with a 61.6% reduction at 10 wt %. Silver nanoparticles enhance conductive pathways and electron transfer, leading to this sharp decline in resistivity.

In contrast, EG-PDA-Ag/VMQ composites maintain good electrical insulation even at higher loadings, with 10 wt % EG-PDA-Ag/VMQ exhibiting a resistivity of 7.03 × 10^9^ Ω·cm, still meeting the standard for electrical insulators (≥10^9^ Ω·cm). This is attributed to the polydopamine (PDA) layer on EG-PDA-Ag, which disrupts the EG’s electron cloud and improves dispersion, preventing the formation of a continuous conductive network. The PDA coating also encapsulates Ag nanoparticles, reducing their conductive effect and further enhancing the insulation. Overall, EG-PDA-Ag/VMQ offers both excellent thermal conductivity and high electrical insulation, making it highly suitable for thermal interface materials in electronic packaging and insulation applications.

### 3.7 Mechanical Properties of VMQ Composites

**Figure 11:**
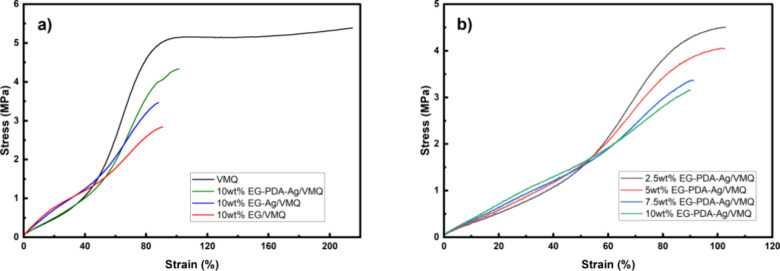
(a) Stress–strain curves of VMQ pure sample and composites with different fillers at 10 wt % filler loading. (b) Stress–strain curves of EG-PDA-Ag/VMQ under different filler loadings.

In order to test the performance of thermally conductive silicone rubber composites in terms of mechanical properties, we performed tensile tests on samples containing 10 wt % filler, and the results are shown in Figure 11. As shown in Figure 11 (a), pure VMQ exhibited the highest tensile strength (5.38 MPa) and strain (216%), while composites with EG, EG-Ag, and EG-PDA-Ag fillers showed reduced tensile strength (2.84, 3.48, and 4.32 MPa, respectively) and strain. However, the EG-PDA-Ag/VMQ composite demonstrated a significant improvement in tensile strength (52.1% and 24.1% higher than EG/VMQ and EG-Ag/VMQ, respectively) and strain due to the synergistic effects of silver nanoparticles and PDA surface modification.

The increased modulus of elasticity in composites is attributed to improved filler dispersion and interfacial adhesion, which restrict polymer chain mobility. However, EG/VMQ composites exhibit lower tensile strength due to weak physical bonding and poor filler dispersion, resulting in stress concentration points. The addition of Ag nanoparticles enhances filler distribution and interfacial bonding, leading to improved tensile strength in EG-Ag/VMQ. PDA modification further enhances tensile strength and strain in EG-PDA-Ag/VMQ through chemical bonding with the VMQ matrix, improved filler dispersion, and reduced agglomeration, which mitigates stress concentration. Additionally, PDA acts as a soft polymer, providing interfacial slip capacity under extreme deformation, absorbing stress, and delaying crack propagation.

Second, as shown in Figure 11**(b**), increased filler content (2.5% to 10%) in EG-PDA-Ag/VMQ results in decreased tensile strength and elongation at break due to reduced flexibility of the VMQ matrix, limited interfacial bonding efficiency, and the formation of a rigid filler network. While this network improves thermal conductivity, it reduces ductility, making the material more prone to fracture under tensile stress.

## 4 Conclusions

In this thesis, a series of VMQ-based composites with thermally conductive fillers—EG, EG-Ag, and EG-PDA-Ag—were developed and evaluated. EG was modified with silver nanoparticles (Ag) through an in situ reduction process and further modified with polydopamine (PDA) to enhance compatibility with the VMQ matrix and reduce electrical conductivity.The results demonstrated that the EG-PDA-Ag/VMQ composite exhibited superior thermal conductivity, electrical insulation, and mechanical properties compared to both EG/VMQ and EG-Ag/VMQ. At 10 wt % filler loading, the EG-PDA-Ag/VMQ composite achieved a thermal conductivity of 1.15 W·m^–1^K^–1^, representing a 638.9% improvement over pure VMQ and a 31% increase over EG-Ag/VMQ. The enhanced thermal conductivity is attributed to improved compatibility and the formation of continuous thermal networks facilitated by the PDA layer and silver nanoparticles.In terms of dielectric properties, EG-PDA-Ag/VMQ showed lower dielectric constant and loss compared to the other composites, making it suitable for applications requiring low dielectric loss. Additionally, it demonstrated excellent electrical insulation with a volume resistivity of 7.03 × 10^9^ Ω·cm, significantly higher than both EG/VMQ and EG-Ag/VMQ.The tensile strength of the EG-PDA-Ag/VMQ composite was also significantly improved, reaching 4.32 MPa—52.1% higher than EG/VMQ and 24.1% higher than EG-Ag/VMQ. These findings suggest that the EG-PDA-Ag/VMQ composite is a promising material for applications that require both high thermal conductivity and electrical insulation. This study demonstrated the excellent thermal, dielectric and mechanical properties of EG-PDA-Ag/VMQ composites, making them ideal candidates for thermal conductivity and electrical insulation applications.

## Supporting Information Available

(Figure S1) Photograph of VMQ and EG-DOPA-VMQ composites and synthesis process of VMQ composites incorporating different fillers; (Figure S2) TGA curves of EG, EG-Ag, and EG-PDA-Ag and FTIR spectra of EG, EG-Ag, and EG-PDA-Ag; (Figure S3) XRD pattern of GO; (Table S1) parameters for Foygel model fitting; (Figure S4) Foygel model fitting of the thermal conductivities of EG/VMQ, EG-Ag/VMQ, and EG-PDA-Ag/VMQ composites; (Figure S5) schematic modeling of thermal conductivity of EG/VMQ, EG-Ag/VMQ, and EG-PDA-Ag/VMQ silicone rubber composites; (Figure S6) comparison of thermal conductivity of 10 wt % EG-PDA-Ag/VMQ with other final composites from other research (PDF)
