# Supplementary material for: Effects of Nanoparticles and Surface Modification on Thermal, Mechanical, and Electrical Properties of Composites from Liquid Silicone Rubber with Expanded Graphite
Source: ACS Omega. 2025 Apr 15;10(16):16370–83. doi: 10.1021/acsomega.4c10633 (PMC12044444; doi:10.1021/acsomega.4c10633)
Supplement: Supplementary file 1 — ao4c10633_si_001.pdf [file ao4c10633_si_001.pdf]

Supporting Infoamation

Effects of nanoparticles and surface modification on thermal, mechanical, and electrical properties of composites from liquid silicone rubber with expanded graphite

Xingrong Liu[a]* , Zhaoyang Ma[a], Dietmar Auhl[a], Fan Xia[b]

*Corresponding Author: Xingrong Liu: Department of Polymer Materials and Technologies, Technische Universität (TU) Berlin, Ernst-Reuter-Platz 1, D-10587 Berlin, Germany; Email: xingrong.liu@campus.tu-berlin.de

Dietmar Auhl: Department of Polymer Materials and Technologies, Technische Universität (TU) Berlin, Ernst-Reuter-Platz 1, D-10587 Berlin, Germany

a Technische Universität (TU) Berlin, Ernst-Reuter-Platz 1, D-10587 Berlin, Germany

b East China University of Science and Technology, Shanghai, 200237, PR China

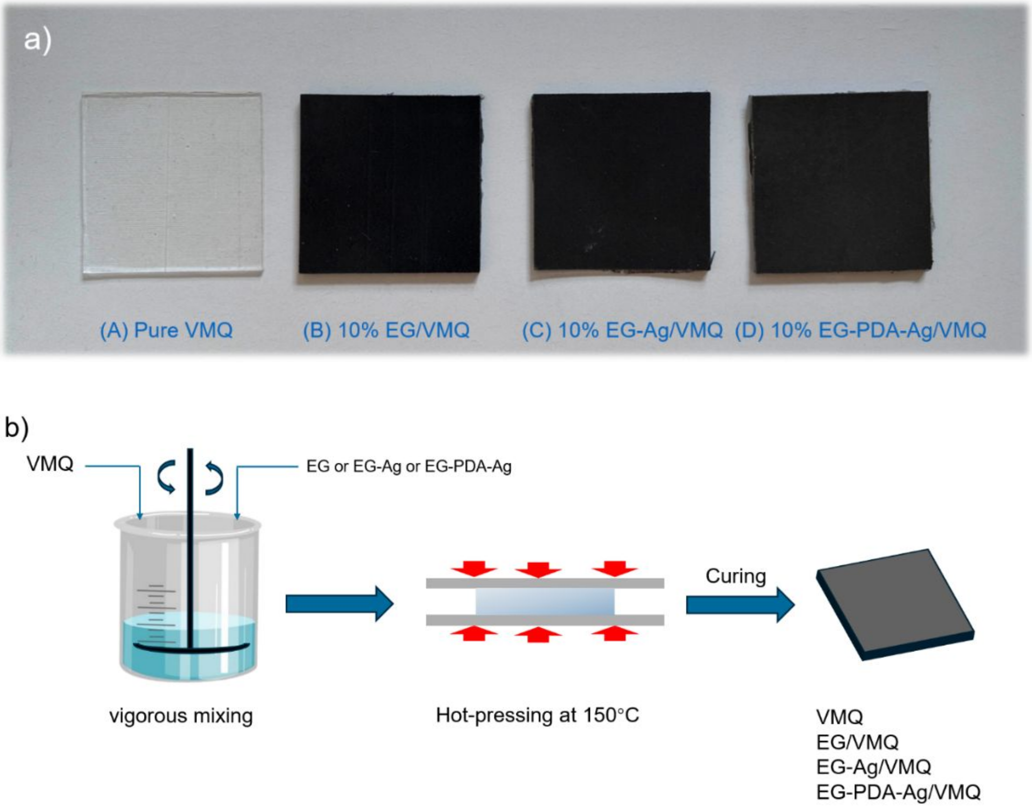

Figure S1. (a) photograph of VMQ and EG-DOPA-VMQ composites.

(b) Synthesis process of VMQ composites incorporating different fillers.

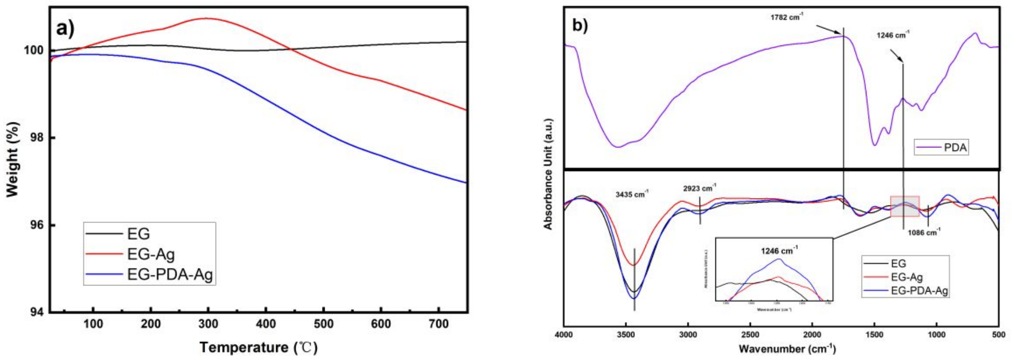

Figure S2. (a)TGA curves of EG, EG-Ag, EG-PDA-Ag (b) FTIR spectra of EG, EG-Ag, EG-PDA-Ag

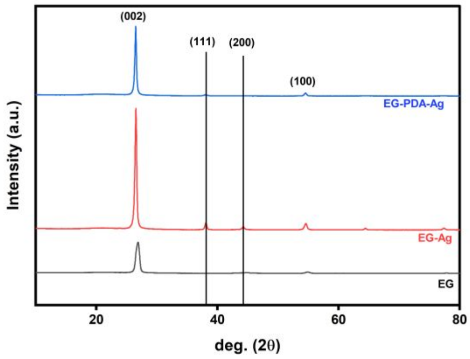

Figure S3. XRD spectra of EG, EG-Ag, EG-PDA-Ag

Table S1 Parameters for fitting the Foygel model

Parameter	EG/VMQ	EG-Ag/VMQ	EG-PDA-Ag/VMQ
L	200 μm	200 μm	200 μm
D	2 μm	2 μm	2 μm

p	100	100	100
f_c	0.6	0.6	0.6
K_0	36.8451	38.4975	40.5968
β	1.09856	1.00091	0.95612
R_c'	4.09869×10^6 KW^{-1}	3.86348×10^6 KW^{-1}	3.66629×10^6 KW^{-1}
A_s	1.34988×10^{-13} m^2	1.34988×10^{-13} m^2	1.34988×10^{-13} m^2
R_c	5.53274×10^{-7} m^2KW^{-1}	5.21523×10^{-7} m^2KW^{-1}	4.94905×10^{-7} m^2KW^{-1}

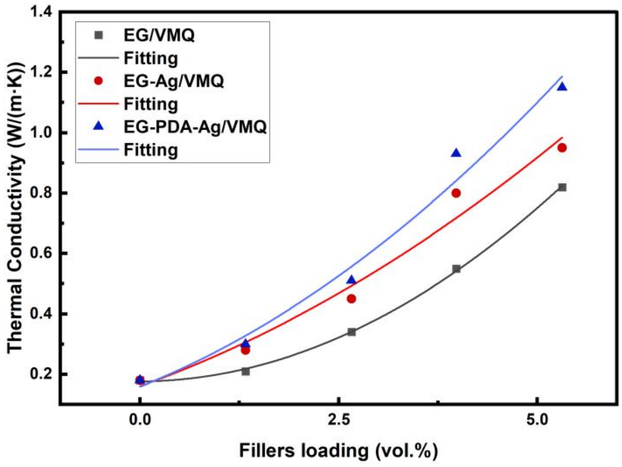

Figure S4 Foygel model fitting of the thermal conductivities of EG/VMQ, EG-Ag/VMQ and EG-PDA-Ag/VMQ composites

(To fit the model, the weight loading fractions of fillers was converted into volume loading fractions)

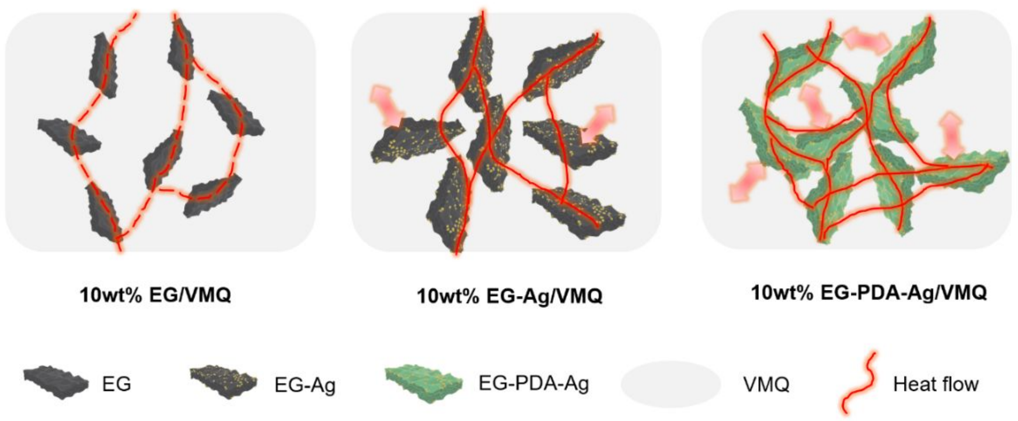

Figure S5. Schematic modeling of thermal conductivity of EG/VMQ, EG-Ag/VMQ and EG-PDA-Ag/VMQ silicone rubber composites.

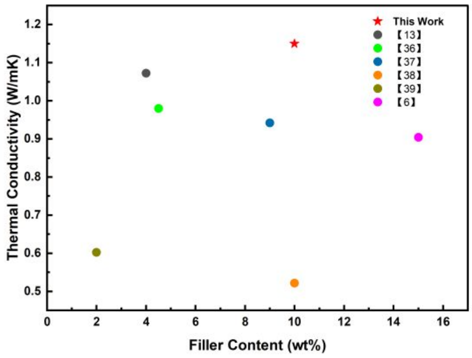

Figure S6. Comparison of thermal conductivity of 10 wt% EG-PDA-Ag/VMQ with other final composites from other research.
